# African American adolescents' experiences providing care for a family member with dementia

**DOI:** 10.1002/alz70858_097581

**Published:** 2025-12-24

**Authors:** Bashir Easter, Janelle Gore, Jarett Fields, Fayron Epps

**Affiliations:** ^1^ Melanin Minded, Milwaukee, WI, USA; ^2^ University of Alabama, Tuscaloosa, AL, USA; ^3^ Brotherhood Health, Houston, TX, USA; ^4^ UT Health San Antonio, San Antonio, TX, USA

## Abstract

**Background:**

In spite of their prevalence, research has overlooked the unique challenges faced by African American adolescent caregivers. This study explores the experiences of African American adolescents aged 14‐17 who provide care for family members with Alzheimer's disease and related dementias (ADRD).

**Method:**

The research utilizes a qualitative approach, conducting semi‐structured interviews with 10 participants recruited through two ADRD service organizations. The theoretical framework integrates Erikson's theory of psychosocial development and Biddle's role theory to examine the internal and external experiences of these adolescent caregivers. Analysis of interview data revealed five key themes: 1) experiencing and recognizing caregiver stress, 2) conflicting roles within the African American family system, 3) knowledge of ADRD behavioral symptoms through direct caregiving experiences, 4) seeking supportive groups instead of traditional support groups, and finally 5) the positive impact of caregiving.

**Result:**

The results of this study show the variety of emotions experienced by these adolescents, including relief, joy, sadness, and stress. Results also highlight the cultural expectations within the families of each caregiver. Notably, participants reported gaining knowledge about ADRD through hands‐on experience rather than formal educational training.

**Conclusion:**

This research addresses a vital gap in the literature and provides valuable insight into the unique needs and experiences of African American adolescent caregivers. The findings can lead to the development of culturally tailored support systems and interventions to better assist adolescent caregivers and their families in managing the unique challenges associated with providing care for a family member with ADRD.

